# Quantitative Relationships between Pulmonary Function and Residual Neuromuscular Blockade

**DOI:** 10.1155/2018/9491750

**Published:** 2018-02-15

**Authors:** ShuYing Fu, WenDong Lin, XiNing Zhao, ShengJin Ge, ZhangGang Xue

**Affiliations:** ^1^Department of Anesthesia, Zhongshan Hospital, Fudan University, Shanghai 200032, China; ^2^Department of Anesthesiology, Shanghai Medical College, Fudan University, Shanghai 200032, China; ^3^Department of Anesthesiology, The Second Affiliated Hospital and Yuying Children's Hospital of Wenzhou Medical University, Wenzhou 325000, China; ^4^Department of Anesthesia, The First Affiliated Hospital of Wenzhou Medical University, Wenzhou 325000, China

## Abstract

**Background:**

Neuromuscular blockade is a risk factor for postoperative respiratory weakness during the immediate postoperative period. The quantitative relationships between postoperative pulmonary-function impairment and residual neuromuscular blockade are unknown.

**Methods:**

113 patients who underwent elective laparoscopic cholecystectomy were enrolled in this study. They all had a pulmonary-function test (PFT) during the preoperative evaluation. Predictive values based on demographic data were also recorded. The train-of-four ratio (TOFR) was recorded at the same time as the PFT and at every 5 minutes in the qualified 98 patients in the postanesthesia care unit (PACU). We analyzed the degree of PFT recovery when the TOFR had recovered to different degrees.

**Results:**

There was a significant difference (*P* < 0.05) between the preoperative baseline value and the postoperative forced vital capacity at each TOFR point, except at a TOFR value of 1.1. There was also a significant difference (*P* < 0.05) between the preoperative baseline value and the postoperative peak expiratory flow at each TOFR point.

**Conclusions:**

Postoperative residual neuromuscular blockade was common (75.51%) after tracheal extubation, and pulmonary function could not recover to an acceptable level (85% of baseline value), even if TOFR had recovered to 0.90.

**Trial Registration:**

Chinese Clinical Trial Register is ChiCTR-OOC-15005838.

## 1. Introduction

Muscle relaxants are widely used in clinical practice to achieve surgical relaxation and to facilitate mechanical ventilation. The clinical objective is for complete recovery from neuromuscular blockade to occur by the time of tracheal extubation. However, on time and complete neuromuscular recovery rarely occurs, even when short- or intermediate-action muscle relaxants, and their reversal agents, are used. This condition may have negative effects on postoperative respiratory function [1].

Various factors are associated with postoperative respiratory function impairment (e.g., patient obesity, surgical incision site, and residual anesthetic agents). Residual neuromuscular blockade (RNMB) is a risk factor for postoperative respiratory weakness during the immediate postoperative period [2, 3].

Pulmonary-function impairment occurs with specific train-of-four ratio (TOFR) values or with RNMB (defined as TOFR < 0.90) [4–6]. However, the quantitative relationships between postoperative pulmonary-function impairment and RNMB are unknown. In this study, we investigated the quantitative relationships between the two variables.

## 2. Materials and Methods

### 2.1. Patient Selection

The study population consisted of patients who underwent elective laparoscopic cholecystectomy at Zhongshan Hospital, Fudan University (Shanghai, China), during 1 February 2015 to 31 May 2015. The study protocol was approved by the Hospital's Ethics Committee (B2014-137) and registered with http://www.chictr.org.cn/: ChiCTR-OOC-15005838. Each patient who participated in the study provided his or her written informed consent.

Inclusion criteria included patients of both genders, 18 to 60 years of age, American Society of Anesthesiologists (ASA) physical status I or II, body mass index (BMI) 20 to 25 kg/m^2^, and who received elective laparoscopic cholecystectomy under general anesthesia. The exclusion criteria included patients with cardiopulmonary dysfunction (e.g., bronchial asthma, chronic obstructive pulmonary disease, and restrictive pulmonary disease), liver or kidney dysfunction, thoracic deformity, and neuromuscular diseases. Patients with a core temperature <36°C in the postanesthesia care unit (PACU) and who were reluctant to undertake the pulmonary-function test (PFT) were excluded during the study period.

### 2.2. Data Collection

During the preoperative evaluation, all participants were familiarized with and underwent a PFT using a spirometer (CareFusion Microlab Jaeger Germany). Three consecutive PFTs with the patient's back reclined at 45° and the knees flexed at 20° to 30° were performed. The highest values for forced vital capacity (FVC) and peak expiratory flow (PEF) were recorded as the baseline PFT result. The predictive values of FVC and PEF, which were calculated using the spirometer result and demographic information, were also recorded. Patients were informed about the discomfort and pain, or both, associated with train-of-four (TOF) measurement (using a TOF-Watch® SX neuromuscular transmission monitor) and the additional PFT that would be performed once during the postoperative period. None of the patients were given premedications.

Standard intraoperative monitoring included electrocardiography (ECG), noninvasive blood pressure (NIBP), pulse oximetry (SpO_2_), and end-tidal carbon dioxide (ETCO_2_) in the operating room. Anesthesia was induced using intravenous anesthetics, including a propofol target-controlled infusion to a plasma concentration of 4 *μ*g·ml^−1^. Intravenous remifentanil (0.2 *μ*g·kg^−1^·min^−1^), fentanyl (2.0–2.5 *μ*g·kg^−1^), rocuronium (0.6 mg·kg^−1^), and lidocaine (1 mg·kg^−1^) were also given. Rocuronium was the unique nondepolarizing agent used for muscle relaxation. Neuromuscular monitoring was not performed during the intraoperative period.

Anesthesia was maintained using intravenous or intravenous compound inhalation anesthesia. A propofol infusion was used for intravenous anesthesia and a sevoflurane inhaled compound propofol infusion was used for compound anesthesia. Fentanyl and rocuronium were infused based on each patient's condition during the intraoperative period. All patients were given flurbiprofen axetil (50 mg) for postoperative pain relief and tropisetron (6 mg) for antiemesis, before wound closure. The anesthesiologists in charge decided whether to reverse neuromuscular function with neostigmine and the extubation time based on clinical signs. The patients were transported into the PACU after extubation.

During postoperative recovery in the PACU, all patients were continuously monitored (ECG, NIBP, SpO_2_), received 5 L/min oxygen via a mask, and were covered with a blanket. The ear temperature of each patient enrolled in the study was measured at arrival to the PACU. Patients with a temperature <36°C were excluded from the study. The TOF-Watch SX was used to immediately test the degree of RNMB (four 0.2 ms pulses at a 2 Hz frequency and 50 mA current intensity at 15 s time interval). The TOFR measurement was repeated three times, and the mean value of the consecutive measurements was recorded. Patients were also checked every 5 min to determine whether they were awake or in pain, had nausea or vomiting, and were willing to perform the PFT. When the patient was willing to perform the test, three consecutive PFT measurements were obtained with the patient's back reclined at 45° and the knees flexed 20° to 30°; the highest of the three values was recorded. If the patient was willing to perform the PFT when TOFR was being measured, PFT measurements were recorded concurrently with TOFR measurements until the patient was discharged from the PACU.

### 2.3. Statistical Analysis

The statistical analysis was performed using IBM SPSS Statistics 19.0 for Windows software (IBM Corp, Armonk, NY). We used the data pairs of TOFR and pulmonary-function values obtained from the PFTs performed at the same time-points during which the TOFRs were measured. We grouped the data pairs into 0.10 intervals, according to the TOFR values. TOFR neuromuscular function was achieved when the recovery point reached an accelerometer measurement value of 0.4 (i.e., 0.40–0.49 range). The other values for each range were 0.5 (0.50–0.59), 0.60 (0.60–0.69), 0.7 (0.70–0.79), 0.80 (0.80–0.89), 0.9 (0.90–0.99), 1.00 (1.00–1.09), and 1.1 (1.10–1.19). We then analyzed the differences in pulmonary function at different TOFR neuromuscular function recovery points. Patients were divided into two groups, RNMB-absent (TOFR ≥ 0.90) and RNMB-present (TOFR < 0.90) based on the TOFR at the first time the PFT was performed. We analyzed the differences between the RNMB-absent and RNMB-present groups to examine the factors that affect RNMB blockade.

Enumerated data were compared using Fisher exact test or chi-square test, as appropriate. Comparison of continuous variables among the groups was performed using one-way analysis of variance with post hoc Bonferroni or Games-Howell tests; if the data are heterogeneity of variance, Kruskal-Wallis test was used. Paired-samples *t*-tests analyzed data within one group. Probability values < 0.05 were indicative of statistically significant differences.

## 3. Results

A total of 113 patients met the inclusion criteria and provided informed consent. Fifteen of these patients were excluded from the study because of pain, nausea, vomiting, a low body temperature, or unwillingness to perform the PFT in the PACU. Ninety-eight patients were identified as the final group of participants. These patients did not have a body temperature <36°C, pain (VSA score ≥ 3), nausea, vomiting, or an SpO_2_ < 98%; they also cooperated willingly with the PFT.

Summary of results for study variables is shown in Table 1. On arrival to the PACU, the mean TOFR was 0.75 ± 0.21; 27.55% (27/98) of the patients had a TOFR < 0.70, and 75.51% (74/98) had a TOFR < 0.90 which was higher than that (65.31%, 64/98) at the first time willing to perform PFT.

Totally, 580 data pairs of TOFR and pulmonary-function values were obtained at different TOFR neuromuscular function recovery points. There was a statistically significant difference (*P* < 0.05) between the preoperative baseline and postoperative FVC value at each TOFR point, except TOFR 1.1. There was a statistically significant difference (*P* < 0.05) between the preoperative baseline value and the postoperative PEF value at each TOFR point (Table 2).

The RNMB-absent and the RNMB-present groups were similar in age, gender, BMI, preoperative pulmonary function, anesthesia maintenance time, fentanyl dosage, and patient-controlled intravenous analgesia ratio. The dosage of rocuronium and the ratio of given muscle relaxant antagonist were significantly different (*P* < 0.05) between the two groups (Table 3).

## 4. Discussion

### 4.1. Postoperative Residual Neuromuscular Blockade

The review of Murphy and Brull [7] revealed that the incidence of RNMB varies from 3.5% to 88%. The meta-analysis of Naguib et al. [8] indicated that the incidence of RNMB is much higher when the TOFR is set as <0.9 and that this value is a more useful diagnostic criterion than a TOFR < 0.7. The incidence of RNMB was also very high in our study population; 27.55% (27/98) of patients had a TOFR < 0.70, and 75.51% (74/98) of them had a TOFR < 0.90 on arrival to the PACU. The patient's age, gender, height, weight, and perioperative conditions may affect neuromuscular function recovery. Postoperative RNMB is more frequent in the elderly (44%) compared with younger (20%) patients [9]. Spontaneous and neostigmine-assisted neuromuscular function recovery is more rapid in children than in adults [10]. Adamus et al. [11] proposed that females are more sensitive to rocuronium. Fujimoto et al. [12] reported that drug pharmacokinetics and pharmacodynamics change in obese patients; muscle relaxation time is significantly prolonged when the drug is administered based on body weight. Wang et al. [13] found that retransfusion of salvaged blood significantly impairs neuromuscular function recovery in the PACU. Teng et al. [14] reported that an elevated PaCO_2_ prolongs spontaneous recovery of the neuromuscular blockade induced by rocuronium. Our study results indicated that age, gender, BMI, preoperative pulmonary function, anesthesia maintenance time, dosage of fentanyl, and ratio of PCIA between the RNMB-absent and the RNMB-present groups were similar, and only the dosage of rocuronium and the ratio of given muscle relaxant antagonist between the two groups were significantly different. That can be explained by our strict inclusion criteria: 18 to 60 years of age, ASA physical status I or II, BMI 20–25 kg/m^2^, and receiving elective laparoscopic cholecystectomy under general anesthesia.

Postoperative pulmonary-function impairment occurred in almost every patient who underwent general anesthesia, especially in patients with RNMB, and other factors include surgical method, artificial pneumoperitoneum, mechanical ventilation, and incomplete metabolism of opioids [2, 15]. Although in PACU no patient had dyspnea, hypoxemia, upper airway obstruction, or bronchial spasm, in our study, they were already exposed to the well-described risks of RNMB.

RNMB can cause airway dysfunction, and impairment of the coordination of pharyngeal and upper esophageal muscles [15]; these conditions can result in airway obstruction, aspiration pneumonia, respiratory failure, bronchospasm, hypoxemia, atelectasis, and other severe postoperative pulmonary complications postoperatively in PACU and/or in the ward. Xara et al. found [16] that RNMB is an independent risk factor for adverse respiratory events in the PACU. Asai and Isono [17] concluded that RNMB after anesthesia is a cause of postoperative aspiration pneumonia. Eikermann et al. [6] found that coordination of upper respiratory muscles was suppressed when neuromuscular function was blocked, even if there were no obvious signs of respiratory insufficiency. Sundman et al. [18] found that partial neuromuscular paralysis is associated with a 4- to 5-fold increase in the incidence of misdirected swallowing. The mechanism of the pharyngeal dysfunction is the delay of the swallowing reflex, which results from the pharyngeal muscle function impairment. Piccioni et al. [19] reported that RNMB in healthy elderly individuals may cause an increased incidence of pharyngeal dysfunction (from 37 to 71%) and impairment of pharyngeal protection of the airway.

Under normal circumstances, pulmonary ventilation increases to reverse hypoxemia and hypercapnia. This response is a protective reflex. In hypoxemic conditions, the RNMB caused by a nondepolarizing muscle relaxant can reduce the sensitivity of the carotid body chemoreceptor and impair the hypoxic ventilation reflex [20].

### 4.2. The Quantitative Relationships between Postoperative Pulmonary Function and Residual Neuromuscular Blockade

FVC reflects the expiratory resistance of the larger airway, and PEF refers to the instantaneous velocity of the expiratory flow during the pulmonary-function test, mainly reflecting the strength of the respiratory muscle and airway obstruction. They can reflect the pulmonary-function reduction caused by RNMB [4, 5]. Eikermann et al. [5] studied 12 healthy volunteers who received a rocuronium injection to induce neuromuscular function decline. When neuromuscular function was severely blocked (TOFR = 0.5), the pulmonary-function data of each of the volunteers was below 90% of the baseline value. When the TOFR recovered to a value of 0.8, the FVC had recovered to 90%, but other pulmonary-function indices had not recovered; when the TOFR was 1.0, only one volunteer's pulmonary function had not recovered. The same authors [6] also performed a similar study of six healthy volunteers monitored with mechanomyography rather than acceleromyography; when the TOFR = 0.5 in these healthy volunteers, the FVC decreased to 89% of the baseline values, and the PEF declined to 80% of the baseline value. Kumar et al. [4] found that the pulmonary-function declines (FVC decreased to 49 ± 18% and PEF to 38 ± 17% of the preoperative baseline values) in the RNMB group were more apparent than those in the non-RNMB group. In our study, there was a significant reduction in the postoperative FVC and PEF values compared with the baseline values (i.e., the greater the RNMB, the greater the reduction). There was a significant difference (*P* < 0.05) between the preoperative baseline value and the postoperative FVC at each TOFR point, except at TOFR 1.1. There was also a significant difference (*P* < 0.05) between the preoperative baseline value and the postoperative PEF at each TOFR point. The study of Kumar et al. [4] results suggested that postoperative pulmonary-function recovery to 85% of the preoperative base value is an acceptable level. In our study, only when TOFR had recovered to more than 1.1, the FVC (0.86 ± 0.09 of preoperative baseline value) could achieve an acceptable level. This may indicate that pulmonary function could not yet return to an acceptable level even though the values obtained during TOFR monitoring of neuromuscular function has recovered to an acceptable level of 0.9.

Our study had some limitations. Firstly, all patients were extubated under the decision of experienced anesthesiologists, and the PFT was performed when the patient was awake and willing to take the test. So, among the 580 data pairs of TOFR and pulmonary-function values, there were only 32 data pairs of TOFR < 0.7. As a result, we could not analyze accurately the correlation of TOFR and pulmonary function. Secondly, the subjects in this study were quite “normal”: 18 to 60 years of age, ASA physical status I or II, BMI 20–25 kg/m^2^, and receiving elective laparoscopic cholecystectomy (less traumatic). Then, the results may not qualify for elderly patients or children, and critical, obese, thin, or patients undergoing major surgeries.

## 5. Conclusion

Our study included a quantitative analysis between postoperative pulmonary function and RNMB. We found that a higher ratio of postoperative RNMB was common (75.51%) after tracheal extubation, and pulmonary function had not recovered to an acceptable level (85% of baseline values), even if TOFR had recovered to 0.90.

## Figures and Tables

**Table 1:** Summary of results for study variables.

	Values
Gender (male/female)	38/60
Age, yrs	48.96 ± 11.22
BMI (kg/m^2^)	23.57 ± 2.34
Preoperative FVC (L)	2.92 ± 0.79
Preoperative PEF (L/s)	6.19 ± 1.87
Ratio of using PCIA	56.12% (55/98)
Ratio of using muscle relaxant antagonist	69.39% (68/98)
Anesthesia time (min)	76.89 ± 24.14
Total dose of rocuronium (mg)	43.98 ± 7.28
Total dose of fentanyl (*μ*g)	231.12 ± 59.97

At the first time willing to perform PFT	
TOFR	0.83 ± 0.17
FVC (L)	2.13 ± 0.65
PEF (L/s)	3.89 ± 1.89

At the time of leaving PACU	
TOFR	0.97 ± 0.07
FVC (L)	2.46 ± 0.63
PEF (L/s)	4.75 ± 1.51

BMI, body mass index; FVC, forced vital capacity; PEF, peak expiratory flow; PCIA, patient-controlled intravenous analgesia; TOFR, train-of-four ratio; PFT, pulmonary-function test; PACU, postanesthesia care unit.

**Table 2:** Postoperative pulmonary function and recovery ratio values at different TOFR neuromuscular function recovery points.

TOFR point	Number of data pairs	Postoperative FVC (L)	Postoperative PEF (L/s)	Recovery ratio of postoperative FVC to basic values	Recovery ratio of postoperative PEF to basic values	Recovery ratio of postoperative FVC to predicted values	Recovery ratio of postoperative PEF to predicted values
0.4	4	1.61 ± 0.42	3.03 ± 0.80	0.61 ± 0.07	0.53 ± 0.08	0.45 ± 0.07	0.42 ± 0.08
0.5	9	1.83 ± 0.41	3.04 ± 0.49	0.63 ± 0.11	0.59 ± 0.10	0.56 ± 0.05	0.47 ± 0.08
0.6	19	2.01 ± 0.48	3.80 ± 1.21	0.70 ± 0.17	0.60 ± 0.17	0.59 ± 0.12	0.52 ± 0.14
0.7	48	2.27 ± 0.56	4.26 ± 1.45	0.76 ± 0.10	0.65 ± 0.15	0.63 ± 0.11	0.57 ± 0.13
0.8	166	2.30 ± 0.57	4.30 ± 1.36	0.79 ± 0.12	0.69 ± 0.17	0.65 ± 0.13	0.59 ± 0.15
0.9	211	2.32 ± 0.66	4.32 ± 1.36	0.83 ± 0.11	0.72 ± 0.16	0.66 ± 0.14	0.60 ± 0.15
1.0	105	2.44 ± 0.64	4.55 ± 1.44	0.84 ± 0.11	0.74 ± 0.17	0.71 ± 0.12	0.64 ± 0.15
1.1	18	2.98 ± 0.54	5.00 ± 1.10	0.86 ± 0.09	0.77 ± 0.13	0.73 ± 0.72	0.65 ± 0.12

FVC, forced vital capacity; PEF, peak expiratory flow.

**Table 3:** Comparison of residual neuromuscular blockade- (RNMB-) present and RNMB-absent groups.

	RNMB-present (*n* = 64)	RNMB-absent (*n* = 34)	*P* value
Age, yrs	48.52 ± 10.67	49.79 ± 12.30	0.296
Gender (male/female)	22/42	16/18	0.220
BMI (kg/m^2^)	23.59 ± 2.43	23.52 ± 2.18	0.462
Anesthesia time (min)	78.20 ± 24.26	74.41 ± 24.08	0.462
Total dose of fentanyl (*μ*g)	235.94 ± 65.29	222.06 ± 47.98	0.278
Total dose of rocuronium (mg)	45.30 ± 7.63	41.32 ± 5.81	0.008
Ratio of using PCIA (yes/no)	35/29	20/14	0.695
Ratio of not using muscle relaxant antagonist	42.19% (27/64)	8.82% (3/34)	0.001
Preoperative FVC (L)	2.94 ± 0.68	2.87 ± 0.98	0.307
Preoperative PEF (L/s)	6.12 ± 1.72	6.33 ± 2.16	0.257

RNMB, residual neuromuscular blockade; BMI, body mass index; FVC, forced vital capacity; PEF, peak expiratory flow; PCIA, patient-controlled intravenous analgesia; TOFR, train-of-four ratio; PFT, pulmonary-function test; PACU, postanesthesia care unit.
